# Grounded in Biology: Why the Context-Dependency of Psychedelic Drug Effects Means Opportunities, Not Problems for Anthropology and Pharmacology

**DOI:** 10.3389/fpsyt.2022.906487

**Published:** 2022-05-11

**Authors:** Stephan Schleim

**Affiliations:** Theory and History of Psychology, Heymans Institute for Psychological Research, Faculty of Behavioral and Social Sciences, University of Groningen, Groningen, Netherlands

**Keywords:** psychedelic drugs, psychedelics, placebo, context-dependency, embodiment, mind-body problem, 4E cognition

## Introduction

Langlitz and colleagues wrote about clinically used psychedelic drugs and the possibility of a “moral psychopharmacology” earlier in this journal (1). They emphasized the context-dependency of the effects of these substances (e.g., ayahuasca, psilocybin) and the importance of understanding their impact on social and moral cognition, particularly now that there is more research on their possible clinical applications (e.g., to facilitate psychotherapy). In this opinion article, I want to, first, reflect on the context-dependency from the perspective of recent research on placebo effects, and, second, clarify different meanings of “moral psychopharmacology”. The latter will be placed in the context of a broader conceived view on drug instrumentalization (2–4) and different values associated with it (5–7). In the conclusion, I will briefly distinguish aspects primarily relevant to theoretical, research-oriented, or applied perspectives, respectively, to inform further theoretical, empirical, and ethical discussion of these topics.

## Context-Dependency of Drugs

In line with the proverbial emphasis on the importance of “set and setting” for the consumption of psychedelic substances, Langlitz and colleagues discuss research reporting different experiences associated with the use of hallucinogens in different social contexts (“settings”). In what has become a classic study, Wallace indeed found that the experiential content of dreams and hallucinations depends on one's cultural and social background (8). Similarly, in another classic study, Bourque and Back found that lower-educed people with lower incomes in the USA described transcendental experiences more in *religious* terms, whereas higher-educated people with higher incomes used more *aesthetic* concepts (9). More recently, a comparison between schizophrenia patients from more or less religious regions in East and West Germany showed that the former reported less religious content in their hallucinations than the latter (10).

Langlitz and colleagues, like other researchers, subsequently distinguish pharmacological and extra-pharmacological effects, particularly in research on psychedelics (1, 11–13). But actually already a classic social-psychological study with adrenaline investigated “cognitive, social, and physiological determinants of emotional state” (14). Subjects either received correct, incorrect, or no information about the expected physiological responses (such as increased heart rate, feeling warmth) of an adrenaline injection presented to them as a vitamin shot.^1^ In a following funny social interaction, they reported significantly more euphoria and were more active *without* the correct information, that is, when they had no rational explanation of the physiological symptoms. A similar outcome was reported for an anger-inducing social interaction. Thus the outcome variable (here: emotional state and behavior) depends on an interaction of the expectation, social setting, and pharmacologically induced physiological state of the subjects.^2^

Research on the placebo effect provides more examples. In recent years, scientists focused on explaining its psychobiological mechanisms and clinical potential (15, 16). Some actually criticize the notion of placebo as an “inert substance” as inconsistent, for something inert can conceptually not have any effects. They thus propose the alternative notions of a “context effect” or “meaning response” (15, 17–20), where the former expression emphasizes social context and the latter term individual beliefs or expectations, thus precisely what Langlitz and colleagues refer to when speaking of extra-pharmacological effects. Strikingly, research has shown that the context effect or meaning response can consist in activating the same physiological pathways as drugs used to alleviate a certain medical problem, such as the activation of endogenous opioids and dopamine for pain treatment (15).

Langlitz and colleagues refer to different epistemic cultures in the natural sciences on the one hand and the humanities and social sciences on the other, with common dichotomies of nature/culture or matter/mind. Consistent with a theoretical framework presented by Greenberg and Bailey, I take the stance which Turkheimer coined “weak biologism” (21, 22). This means that there is no strict dichotomy between the biological and non-biological, because in some sense *all* of our perceptions, thoughts, behaviors, and the like are biological—if only in that they have a biological basis, because we are *embodied* beings (23–27). These bodies were shaped through an evolutionary history and particularly their nervous systems enable a wide range of psychological and cultural possibilities, which in turn also influence biological structure and function (i.e. neuroplasticity). For example, certain brain (and other physiological) structures allow us to acquire language; without them, we could not understand and express it. But it's the psychosocial context determining whether one's primary language will be, say, Chinese, English, or Spanish.

Applied to Langlitz's and colleagues' thoughts, this means that there is also no strict dichotomy between pharmacological and extra-pharmacological factors: Just as anthropological research-at least implicitly—*always* includes (and presupposes) certain bodies and brains, pharmacological research *always* includes (and presupposes) a psychosocial context, often a clinical setting in which a substance is administered. In other words, our cognitive and emotional processes are not only (physically) *embodied*, but also (psychosocially) *embedded* (23–27). Kaptchuk already described how, from an anthropological perspective, common procedures even in Western medical systems could be described as a “ritual”, thus a particular psychosocial context affecting the treatment effects (28).^3^ We may tend to overlook this, because we take that context for granted. It then also makes sense that research on psychedelic substances in particular prompts scholars to focus more on context effects, as these substances (like ayahuasca, religiously used in South America and consisting of *N,N*-Dimethyltryptamine [DMT] and a monoamine oxidase inhibitor [MAOI]) often originate in different cultures with particular practices of consumption or “rituals” (13, 29–31).

The upshot of my proposal is that there is no intrinsic contradiction between the tasks of anthropologists, psychologists, or pharmacologists: The latter often won't investigate the psychosocial context (as an independent variable, that is) simply for the reason that the respective drug is supposed to be taken *within* a particular context. Thus, in principle, if pharmacologists became interested in cultural differences or specific effects of psychosocial contexts, they could simply include them in their experimental designs. Whether the resulting discipline deserved a new denomination like “transcultural pharmacology” (cf. transcultural psychiatry) or “pharmanthropology” lies in the eye of the beholder. Strictly distinguishing pharmacological and extra-pharmacological effects, by contrast, carries the risk of reintroducing unnecessary dichotomies; unnecessary, because, as we have seen, the former never comes without the latter.

One could speculate, though, whether the biological (or pharmacological) domain has some primacy in the sense that without the enabling physiological structures and functions there would simply be no anthropological, social, or psychological domain. Experimentally this could be exemplified—within ethical boundaries—by increasing pharmacological doses and thus eventually overriding or at least minimizing effects of psychosocial context (32). Anesthesia is a clear example, as it transiently disables certain psychosocially necessary functions—and thus psychosocial processes. In the words of a patient participating in one of Delgado's early brain stimulation experiments: “I guess, Doctor, that your electricity is stronger than my will” (33, 34). But this does not make the case for “strong biologism”, on Turkheimer's account, which would mean that psychosocial functions could be explained completely or at least for a large part in biological terms. Similar to how Steven Hyman, former director of the US National Institute of Mental Health, recently characterized psychiatric disorders, one could say: The psychosocial processes of pharmaceutical drugs are *grounded*, but not exhausted in biology (35).

## Moral Psychopharmacology and Values

Langlitz and colleagues continue to discuss a possible “moral psychopharmacology”. Indeed, after neuroscientists began to investigate moral decision-making (36–40), pharmacologists also addressed that domain (41, 42) and ethicists speculated about “moral enhancement”, the possibility of using drugs to improve people's moral capacities (43, 44). The ecological validity of the moral dilemmas often used in such studies and their (alleged) social implications have subsequently been discussed critically (45–48). Here it helps, in my view, to distinguish different meanings of “moral” on the one hand and individual or collective perspectives on morality on the other.

In a loose sense of “moral”, as pertaining to *moral implications*, the term is more or less equivalent with “social”. Then, according to Langlitz's and colleagues' call for more awareness for the potential effects of psychedelic drugs in the social domain, one could say that this is valid for psychoactive drugs in general. Unless one imagines the life of a hermit, it would be difficult to think of a psychopharmacological application that could not, in principle, have *any* social implications (e.g., think of the possibility of substances to interfere with people's capacity to control vehicles or machines, which could in turn harm themselves or others and thus become socially relevant). Here it would be important to distinguish transient and permanent effects (with the latter possibly altering personality). It would still make sense, as Langlitz and colleagues suggest, to investigate the potential effects of psychedelic (and other psychoactive) drugs on social cognition. But this should be distinguished, in my view, from a “moral psychopharmacology” in a narrower sense: that is, one that specifically aims at improving subjects' moral cognition. To my knowledge, pharmacological experiments so far used the previously mentioned moral decision-making paradigms, but did not explicitly try to enhance moral competence as operationalized by, for example, Lind's Moral Judgment Test (49, 50). The general problem remains that there is no single accepted standard of what a “good” moral decision is; this would always presuppose a particular moral theory or stance, of which there are many different ones competing in moral philosophy.

Before improving moral capacities, there thus has to be a value judgment on what kind of moral capacity is deemed desirable. Langlitz and colleagues, just like Evers before, particularly address the possibility of increasing empathy on the neurobiological level (1, 51–53). But this raises the question whether more empathy is *always* morally good. It could make people prone to overrate the preferences of others at the cost of their own wellbeing (54). What if egoists or “successful psychopaths” who hardly care for the interests of others as a value in itself disagree to become “morally enhanced” in this sense? This also raises the question *who* is to decide: Without informed consent of the subjects themselves, some would consider “moral psychophamarcology” as coercive or even totalitarian. And based on informed consent there would probably always be individuals deciding *against* such an intervention, simply because people have different values and think differently about the instrumental use of substances. This dilemma was actually already debated by psychiatrist Klerman and bioethicist Veatch half a century ago (5–7). Different value systems and different understandings of human nature shape people's views on drug use differently. The category not addressed by Langlitz and colleagues, but central in medical ethics, is that of *autonomy*. People should primarily decide for themselves, but also considering their own psychosocial context (55). From this perspective, “moral psychopharmacology” could be understood as one example of instrumental substance use more broadly conceived (2–4). People might use drugs to become more moral—*if and only if* they themselves desire so.

## Conclusion

On the theoretical level, psychosocial effects (contexts and beliefs/expectations) on how a drug (psychedelic or not) work are neither surprising nor an insurmountable problem. When humans are conceived as a psychobiological unity in a social environment, “weak biologism” or the idea of the psychosocial domain being *grounded* in biology predicts precisely that: that psychological processes are *embodied* and thus also reflected in a subject's physiological state (23–27). An example from research on the placebo effect has shown that the physiological pathway can actually be the same as the one activated by a drug to treat a particular medical problem (15). This also exemplifies the clinical relevance of understanding the effects of context or beliefs/expectations on drugs. Generally speaking, there is no reason why anthropologists should disregard pharmacology or why pharmacologists should neglect psychosocial context—*if* that becomes salient for their research questions. Whether this justifies the establishment of a “transcultural pharmacology” or “pharmanthropology” as an independent sub-discipline may be a matter of taste. But from the point of view presented here, the context-dependency of psychedelic drug effects rather means opportunities, not problems for anthropology and pharmacology.

## Author Contributions

The author confirms being the sole contributor of this work and has approved it for publication.

## Funding

This publication has been supported by the History of Neuroethics grant by the Dutch Research Foundation (NWO), Grant Number: 451-15-042.

## Conflict of Interest

The author declares that the research was conducted in the absence of any commercial or financial relationships that could be construed as a potential conflict of interest.

## Publisher's Note

All claims expressed in this article are solely those of the authors and do not necessarily represent those of their affiliated organizations, or those of the publisher, the editors and the reviewers. Any product that may be evaluated in this article, or claim that may be made by its manufacturer, is not guaranteed or endorsed by the publisher.

## Footnotes

^1^It goes without saying that, from a present perspective, this procedure is ethically dubious. Yet the study is so informative that it already received more than 10,000 Google Scholar citations and is still cited frequently.

^2^It should be noted, though, that their placebo control was descriptively, but not statistically significantly between the conditions with and without the correct information. This should be seen in the context of the small sample sizes per group (*N* = 25 or 26).

^3^A peer reviewer made the interesting remark that purported effects of microdosing of psychedelic drugs in the newly arising microdosing subculture has topical relevance and may be interesting in the context of discussing placebo effects and research Kaptchuck's. Further pursuing this link goes beyond the scope of this short opinion article.
